# Supplementary material for: The genome sequence of the Southern Grizzled Skipper, Pyrgus malvoides (Elwes & Edwards, 1897) (Lepidoptera: Hesperiidae)
Source: Wellcome Open Res. 2026 Aug 25;10:404. Originally published 2025 Jul 31. [Version 2] doi: 10.12688/wellcomeopenres.24653.2 (PMC12780514; doi:10.12688/wellcomeopenres.24653.2)
Supplement: Peer review history

# Reviewer report: Reviewer response for version 1

**DOI:** 10.21956/wellcomeopenres.27167.r136210

**Published:** 2026-06-11

**Authors:** Gulab D Khedkar

**Affiliations:** 1 Dr. Babasaheb Ambedkar Marathwada University, Aurangabad, Maharashtra, India

**Manuscript review** **report**

 This manuscript presents a high-quality chromosome-level genome assembly of *Pyrgus malvoides*, generated using state-of-the-art PacBio HiFi and Hi-C sequencing technologies. The study provides a valuable genomic resource for a species of considerable evolutionary and taxonomic interest, particularly given its close relationship with *Pyrgus malvae* and the ongoing questions surrounding species divergence and hybridization.

 The work is technically rigorous and demonstrates excellent assembly quality, with nearly complete chromosomal assignment, high BUSCO completeness, and outstanding consensus accuracy. The authors have employed a comprehensive assembly, curation, and validation workflow that meets and exceeds current Earth BioGenome Project standards. The manuscript is clearly written, methodologically transparent, and provides unrestricted access to all underlying datasets and analytical resources, thereby maximizing its value to the scientific community.

 Overall, this study represents a significant contribution to Lepidopteran genomics and will serve as an important reference for future investigations in evolutionary biology, comparative genomics, taxonomy, and conservation genetics

Are sufficient details of methods and materials provided to allow replication by others?

Yes

Is the rationale for creating the dataset(s) clearly described?

Yes

Are the datasets clearly presented in a useable and accessible format?

Yes

Are the protocols appropriate and is the work technically sound?

Yes

Reviewer Expertise:

Genomics, population genetics

I confirm that I have read this submission and believe that I have an appropriate level of expertise to confirm that it is of an acceptable scientific standard.

# Reviewer report: Reviewer response for version 1

**DOI:** 10.21956/wellcomeopenres.27167.r142801

**Published:** 2026-01-10

**Authors:** Luana Bataglia

**Affiliations:** 1 Universita Cattolica del Sacro Cuore - Campus di Piacenza e Cremona (Ringgold ID: 96985), Piacenza, Emilia-Romagna, Italy

The article reports the genome sequencing and assembly of the species *Pyrgus malvoides. *The article is sufficient as a data note, providing complete technical information. I have some suggestions for complementing the study as follows: 

 1) The background is a bit difficult to follow because there is no linearity in the ideas. I suggest that the authors focus less on biological aspects and more on the importance of the sequencing for the species, as well as the contribution of the study. 

 2) There is no information about gene annotation. I strongly suggest that the authors perform it and include it in the article to make it more complete.

 3) The authors mention in the background the possibility of two subspecies for *P. malvoides* (ssp. *malvoides* and ssp. *modestior*) supported by mitochondrial DNA analysis, but in the results, they don't mention to which subspecies the sequenced specimen belongs, and if the resulting sequencing supports this division. This data could be interesting to report.

Are sufficient details of methods and materials provided to allow replication by others?

Yes

Is the rationale for creating the dataset(s) clearly described?

Yes

Are the datasets clearly presented in a useable and accessible format?

Yes

Are the protocols appropriate and is the work technically sound?

Yes

Reviewer Expertise:

Molecular Biology, Genetics, Epigenetics, Entomology, Biotechnology.

I confirm that I have read this submission and believe that I have an appropriate level of expertise to confirm that it is of an acceptable scientific standard.

# Reviewer report: Reviewer response for version 1

**DOI:** 10.21956/wellcomeopenres.27167.r142796

**Published:** 2026-01-08

**Authors:** Taslima Sheikh

**Affiliations:** 1 Baba Ghulam Shah Badshah University, Rajouri, Jammu and Kashmir, India

This manuscript presents a high-quality, chromosome-level genome assembly of *Pyrgus malvoides* using PacBio HiFi and Hi-C data. All methodological, analytical, ethical, and data-availability criteria are fully satisfied, and the results are scientifically sound and well supported. No points require correction for scientific validity; only minor editorial revisions are suggested. Authors have done good work

Are sufficient details of methods and materials provided to allow replication by others?

Yes

Is the rationale for creating the dataset(s) clearly described?

Yes

Are the datasets clearly presented in a useable and accessible format?

Yes

Are the protocols appropriate and is the work technically sound?

Yes

Reviewer Expertise:

lepidoptera

I confirm that I have read this submission and believe that I have an appropriate level of expertise to confirm that it is of an acceptable scientific standard.

# Reviewer report: Reviewer response for version 1

**DOI:** 10.21956/wellcomeopenres.27167.r142800

**Published:** 2026-01-07

**Authors:** Matthew Merkin

**Affiliations:** 1 University of Liverpool, Liverpool, UK

The authors present a chromosome-level genome assembly of the European butterfly *Pyrgus malvoides* (The Southern Grizzled Skipper) as part of Project Psyche. An adult male specimen was collected in Switzerland and sequenced to obtain both HiFi and HiC reads for assembly and scaffolding. Manual correction and quality assessment then took place, with the primary haplotype possessing an EBP score of 6.7.Q67 and BUSCO completeness of 98.6%; these suggest that the genome is of a high standard and suitable for use as a reference genome. 

 This report follows the same format as all Project Psyche genome notes, so I only have two minor comments:

 The introduction is of outstanding quality, which clearly highlights the importance of producing a genome assembly for this species being created and a useful suggestion of how the genome can be used. However, there are many irrelevant details, such as a long description of their sheltering behaviour. Whilst these details are certainly interesting, they make the introduction unnecessarily long and less focussed.

 Table 3 has assigned the largest chromosome (OZ183547.1) as a fusion between M2 and M14. However, the busco painting in Figure 3 suggests that it is only M2, with M14 being on the 22nd largest chromosome (OZ183569.1).

Are sufficient details of methods and materials provided to allow replication by others?

Yes

Is the rationale for creating the dataset(s) clearly described?

Yes

Are the datasets clearly presented in a useable and accessible format?

Yes

Are the protocols appropriate and is the work technically sound?

Yes

Reviewer Expertise:

comparative genomics, entomology, genome assembly, museomics

I confirm that I have read this submission and believe that I have an appropriate level of expertise to confirm that it is of an acceptable scientific standard.

# Reviewer report: Reviewer response for version 1

**DOI:** 10.21956/wellcomeopenres.27167.r135243

**Published:** 2025-11-10

**Authors:** Olli-Pekka Smolander

**Affiliations:** 1 University of Helsinki, Helsinki, Finland

This is an exemplary, high-quality data note that is logical, consistent, and scientifically rigorous. The rationale for generating the genome is clearly articulated: *Pyrgus malvoides* has a complex taxonomic relationship with its sister species, *Pyrgus malvae*, with which it is known to hybridize. This genome provides an important and high-quality resource for comparative genomics to investigate these evolutionary questions and to explore existing evidence of cryptic diversity within the species.

 The work is technically exceptional, employing a state-of-the-art methodology combining high-accuracy PacBio HiFi long reads for the primary assembly with Hi-C data for scaffolding and phasing. The authors' attention to detail is evident, the work resulting in a fully phased, diploid assembly.

 The primary haplotype is 746.71 Mb, with 99.8% of the sequence anchored into 31 chromosomal pseudomolecules. The assembly's quality is demonstrated by a very high contig N50 (9.82 Mb), high accuracy (QV score of 67.7), and high completeness (98.6% complete BUSCOs). This work meets and exceeds the rigorous Earth BioGenome Project (EBP) reference standard.

 The methods are documented with commendable transparency, providing sufficient detail on all materials, specimen data, and laboratory protocols to ensure replication. This commitment to open science extends to their computational methods, with links provided to their curation process in a public Git repository and to the pipelines used. The inclusion of a complete list of all software and their specific versions further highlights the project's rigorous approach. Furthermore, the presentation of results is enhanced by links to interactive versions of the key quality plots, such as those from BlobToolKit Finally, the datasets are clearly presented and made fully accessible in public repositories (ENA project PRJEB79796) and are slated for presentation in the Ensembl genome browser, ensuring their widespread and long-term utility for the scientific community.

Are sufficient details of methods and materials provided to allow replication by others?

Yes

Is the rationale for creating the dataset(s) clearly described?

Yes

Are the datasets clearly presented in a useable and accessible format?

Yes

Are the protocols appropriate and is the work technically sound?

Yes

Reviewer Expertise:

Genomics, multiomics, bioinformatics, computational biology, machine learning, microbiome

I confirm that I have read this submission and believe that I have an appropriate level of expertise to confirm that it is of an acceptable scientific standard.
